# The DEK oncogene activates VEGF expression and promotes tumor angiogenesis and growth in HIF-1α-dependent and -independent manners

**DOI:** 10.18632/oncotarget.8060

**Published:** 2016-03-14

**Authors:** Yanan Zhang, Jie Liu, Shibin Wang, Xiaoli Luo, Yang Li, Zhaohui Lv, Jie Zhu, Jing Lin, Lihua Ding, Qinong Ye

**Affiliations:** ^1^ Department of Medical Molecular Biology, Beijing Institute of Biotechnology, Collaborative Innovation Center for Cancer Medicine, Beijing, People's Republic of China; ^2^ Institute of Cancer Stem Cell, Cancer Center, Dalian Medical University, Liaoning, People's Republic of China; ^3^ First Affiliated Hospital, Chinese PLA General Hospital, Beijing, People's Republic of China; ^4^ Department of Endocrinology, Chinese PLA General Hospital, Chinese PLA Medical School, Beijing, People's Republic of China

**Keywords:** angiogenesis, VEGF, DEK, HIF-1α, tumor growth

## Abstract

The *DEK* oncogene is overexpressed in various cancers and overexpression of DEK correlates with poor clinical outcome. Vascular endothelial growth factor (VEGF) is the most important regulator of tumor angiogenesis, a process essential for tumor growth and metastasis. However, whether DEK enhances tumor angiogenesis remains unclear. Here, we show that DEK is a key regulator of VEGF expression and tumor angiogenesis. Using chromatin immunoprecipitation assay, we found that DEK promoted *VEGF* transcription in breast cancer cells (MCF7, ZR75-1 and MDA-MB-231) by directly binding to putative DEK-responsive element (DRE) of the *VEGF* promoter and indirectly binding to hypoxia response element (HRE) upstream of the DRE through its interaction with the transcription factor hypoxia-inducible factor 1α (HIF-1α), a master regulator of tumor angiogenesis and growth. DEK is responsible for recruitment of HIF-1α and the histone acetyltransferase p300 to the VEGF promoter. DEK-enhanced VEGF increases vascular endothelial cell proliferation, migration and tube formation as well as angiogenesis in the chick chorioallantoic membrane. DEK promotes tumor angiogenesis and growth in nude mice in HIF-1α-dependent and -independent manners. Immunohistochemical staining showed that DEK expression positively correlates with the expression of VEGF and microvessel number in 58 breast cancer patients. Our data establish DEK as a sequence-specific binding transcription factor, a novel coactivator for HIF-1α in regulation of *VEGF* transcription and a novel promoter of angiogenesis.

## INTRODUCTION

Angiogenesis plays a critical role in the development and progression of cancer since adequate blood supply is necessary for cancer cell growth, invasion and metastasis [1]. Vascular endothelial growth factor (VEGF) (also known as VEGF-A), a key regulator of angiogenesis, is a dimeric glycoprotein secreted by many types of cells, including cancer cells, peripheral blood mononuclear cells, and fibroblast cells, but usually not endothelial cells [2–5]. VEGF is important not only for proliferation and migration of endothelial cells but also for proliferation and/or survival of solid cancer cells in an autocrine and/or paracrine manner. VEGF is overexpressed in various human cancers and high VEGF expression associates with short disease-free and overall survival in various cancers. Thus, VEGF is an attractive target for cancer therapy.

Cellular hypoxia is an important phenomenon in cancer [6]. As a transcription factor, hypoxia-inducible factor 1α (HIF-1α) is a master regulator responsible for the induction of genes that assist cancer cells to survive and metastasize from normoxia to hypoxia [7, 8]. HIF-1α, a hypoxia-inducible protein, forms a heterodimer with HIF-1β, which is constitutively expressed. The heterodimeric complex binds to hypoxia response element (HRE) upstream of hypoxia-regulated genes, modulating expression of a variety of HIF-1 target genes, including *VEGF*. Increased tumor HIF-1α is correlated with increased angiogenesis, aggressive tumor growth, and poor patient prognosis, leading to the current interest in HIF-1α as a cancer drug target [9, 10]. However, chemotherapeutic drugs targeting VEGF and HIF-1α have limited efficacy against cancer and even cause adverse effects, suggesting that the mechanisms of VEGF- and HIF-1α-related function need to be further elucidated.

The oncoprotein DEK, a non-histone chromosomal factor, was originally identified as a fusion protein with the CAN nucleoporin in a subtype of acute myeloid leukemias [11]. DEK is overexpressed in many types of solid cancers [12–20]. High expression of DEK associates with poor prognosis of various cancers [21–25], such as breast cancer and prostate cancer. DEK has two distinct DNA-binding domains, a C-terminal DNA binding domain and a SAP-box, a sequence motif that DEK shares with a number of other chromatin proteins [26]. Although some studies show that DEK can directly bind DEK-responsive element (DRE), whether DEK binds to sequence-specific DNA is controversial [27–33]. Overexpression of DEK promotes epithelial transformation and tumor growth [34, 35]. Since VEGF is a key regulator of tumor angiogenesis under normoxic and hypoxic conditions, we screened a transcription factor array, and identified DEK as a regulator of VEGF expression for the first time. Further functional analysis showed that DEK promotes tumor angiogenesis via VEGF. Mechanistically, DEK enhances VEGF transcription by directly binding to the DRE of VEGF promoter and indirectly binding to the HRE upstream of the DRE through its interaction with HIF-1α.

## RESULTS

### DEK increases VEGF expression in breast cancer cells

**Figure 1:**
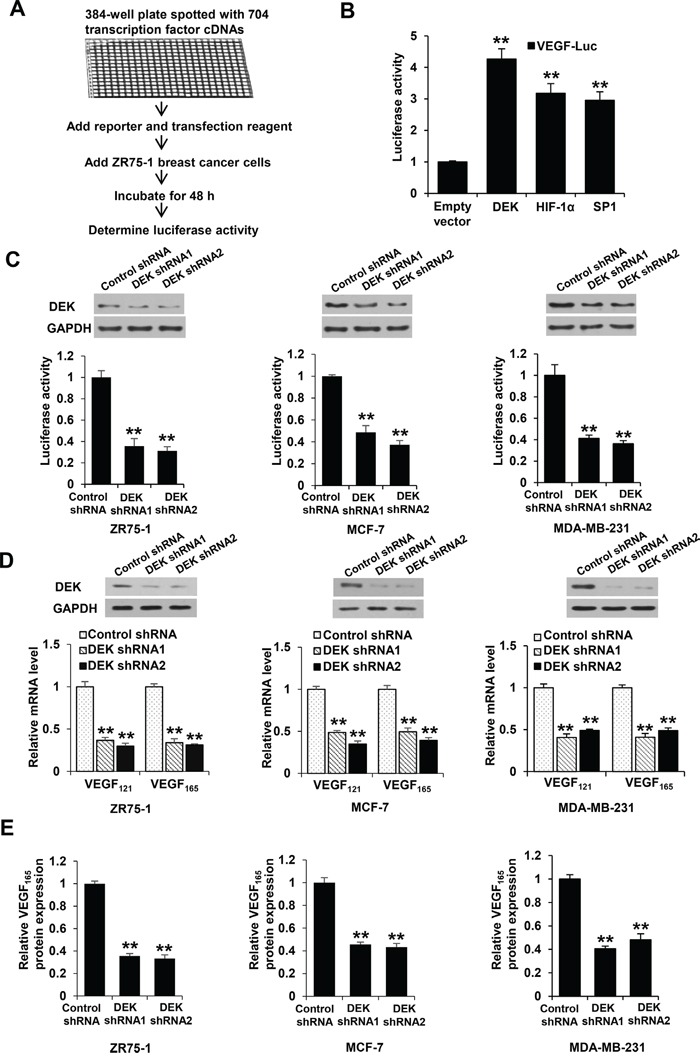
DEK modulates VEGF expression in breast cancer cells **A.** Schematic demonstration of screening for transcription factors that regulate VEGF-Luc reporter activity in ZR75-1 breast cancer cells. **B.** ZR75-1 breast cancer cells were cotransfected with the VEGF-Luc reporter and the indicated transcription factors from A. Luciferase reporter assays were performed to detect the effect of transcription factors on VEGF-luc reporter activity. All values shown are expressed as the mean ± SD obtained from two independent experiments. **P < 0.01 versus empty vector. **C.** ZR75-1, MCF-7 and MDA-MB-231 breast cancer cells were cotransfected with VEGF-Luc and DEK shRNA1, DEK shRNA2 or control shRNA. Luciferase reporter assays were performed to detect the effect of DEK shRNAs on VEGF-luc reporter activity. Representative immunoblot indicates the expression of DEK. GAPDH was used as a loading control. **D.** Real-time RT-PCR analyses of the expression of VEGF_121_ and VEGF_165_, two major VEGF isoforms, in ZR75-1, MCF-7, and MDA-MB-231 cells stably infected with lentivirus carrying DEK shRNAs or control shRNA. Representative immunoblot shows DEK expression. **E.** VEGF concentration in cell supernatants from ZR75-1, MCF-7 and MDA-MB-231 cells stably infected as in D was analyzed by ELISA assay. Data shown are mean ± SD of triplicate measurements that have been repeated 3 times with similar results (C-E). *P < 0.05, **P < 0.01 versus control shRNA.

To identify previously unreported transcription factors that regulate *VEGF* transcription, we used *VEGF* promoter (from −2304 to +73 bp) fused with a gene encoding luciferase as a reporter (VEGF-Luc) to screen a transcription factor genome-wide full-length cDNA-transfection (GFC-transfection) array, consisting of 704 transfection-ready cDNA plasmids, and identified some transcription factors that stimulated the reporter gene expression in ZR75-1 breast cancer cells (Figure 1A, 1B; data not shown), such as DEK and the previously reported transcription factors SP1 and HIF1α [36, 37]. We further confirmed DEK overexpression-mediated enhancement of VEGF-Luc reporter activity using our DEK expression construct in ZR75-1, MCF-7 and MDA-MB-231 breast cancer cells (Supplementary Figure S1A). In contrast, knockdown of DEK with DEK shRNA1 or DEK shRNA2 decreased VEGF-Luc reporter activity in these cells (Figure 1C). Consistent with the results of the luciferase reporter analysis, DEK overexpression enhanced *VEGF* mRNA expression (Supplementary Figure S1B) and VEGF secretion level (Supplementary Figure S1C), whereas DEK knockdown decreased *VEGF* mRNA expression (Figure 1D) and secretion of endogenous VEGF protein (Figure 1E).

### DEK enhances VEGF expression in HIF-1α-dependent and -independent manners

**Figure 2:**
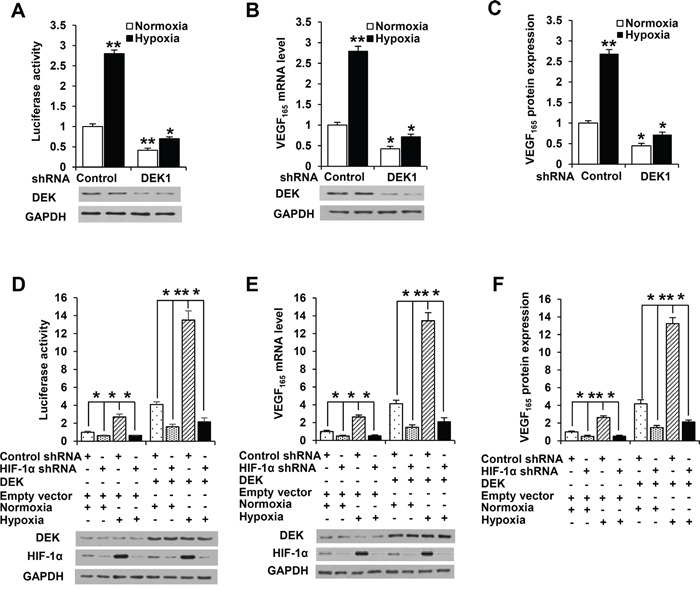
DEK controls VEGF expression in HIF-1α-dependent and -independent manners **A.** Luciferase reporter assays in MCF-7 breast cancer cells cotransfected with the VEGF-Luc reporter and DEK shRNA1 or control shRNA. At 24 h post-transfection, cells were exposed to either normoxic (20% O_2_) or hypoxic (1% O_2_) conditions for another 24 h. Cells were harvested for determination of VEGF-Luc reporter activity. Representative immunoblot shows the expression of DEK. **B.** Real-time RT-PCR analysis of VEGF_165_ expression in MCF-7 cells stably infected with lentivirus carrying DEK shRNA1 or control shRNA. Cells were exposed to either normoxic (20% O_2_) or hypoxic (1% O_2_) conditions for 24 h before collected for immunoblot. Representative immunoblot indicates the expression of DEK. **C.** ELISA analyses of the VEGF concentration in cell supernatants from MCF-7 cells stably infected and treated as in B. **D.** Luciferase reporter assays in MCF-7 cells cotransfected with the VEGF-Luc reporter, DEK and HIF-1α shRNA as indicated. Cells were treated and analyzed as in A. Representative immunoblot shows the expression of DEK and HIF-1α. **E.** Real-time RT-PCR was performed to analyze VEGF_165_ expression in MCF-7 cells stably infected with lentivirus carrying DEK and HIF-1α shRNA as indicated. Cells were treated and analyzed as in B. Representative immunoblot shows the expression of DEK and HIF-1α. **F.** ELISA assay was performed to analyze VEGF concentration in cell supernatants from MCF-7 cells stably infected and treated as in E. All values shown are mean ± SD of triplicate measurements and have been repeated 3 times with similar results. The average of triplicate measurements of the control under normoxia condition was set as 1, and all other groups were compared with the control under normoxia condition. *P < 0.05, **P < 0.01 versus control shRNA or control shRNA plus empty vector.

Since hypoxia is a key phenomenon in cancers [6], we tested whether DEK has a role in regulation of *VEGF* promoter activity and expression under hypoxic conditions using luciferase reporter assay, real-time reverse transcription-PCR (RT-PCR) and enzyme-linked immunosorbent assay (ELISA). As expected, hypoxia enhanced VEGF-Luc reporter activity. Importantly, knockdown of DEK greatly reduced hypoxia-mediated VEGF-Luc reporter activity (Figure 2A; Supplementary Figure S2A). Consistent with the results of the luciferase reporter analysis, DEK knockdown greatly decreased hypoxia-mediated *VEGF* mRNA expression (Figure 2B; Supplementary Figure S2B) and VEGF secretion level (Figure 2C; Supplementary Figure S2C). In addition, DEK knockdown also inhibited VEGF-Luc reporter activity, *VEGF* mRNA expression, and VEGF secretion level (Figure 2A-2C; Supplementary Figure S2A-S2C).

As HIF-1α is a master regulator of VEGF expression in response to hypoxia, we determined whether DEK modulation of *VEGF* promoter activity and expression depends on HIF-1α using luciferase reporter assay, RT-PCR and ELISA. As expected, knockdown of HIF-1α reduced VEGF-Luc reporter activity, *VEGF* mRNA expression, and VEGF secretion level (Figure 2D-2F; Supplementary Figure S2D-S2F). Intriguingly, HIF-1α knockdown greatly reduced but not abrogated DEK-mediated enhancement of VEGF-Luc reporter activity, *VEGF* mRNA expression and VEGF secretion level (Figure 2D-2F; Supplementary Figure S2D-S2F). DEK did not alter the expression of HIF-1α. These data suggest that DEK increases VEGF expression in HIF-1α-dependent and -independent manners.

### Cancer cell-secreted VEGF modulated by DEK controls human umbilical vascular endothelial cell (HUVEC) proliferation and migration

**Figure 3:**
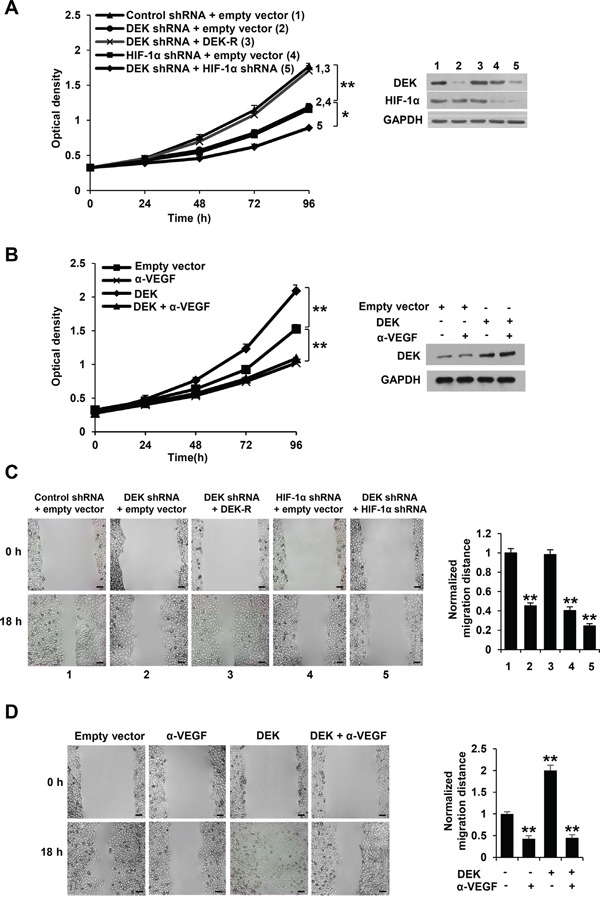
Cancer cell-secreted VEGF modulated by DEK regulates HUVEC proliferation and migration **A.** Cell proliferation assays were performed in HUVEC cells cultured in conditioned medium from MCF-7 cells stably infected with lentivirus carrying DEK shRNA, DEK shRNA plus DEK-resistant shRNA (DEK-R), HIF-1α shRNA or DEK shRNA plus HIF-1α shRNA under hypoxic condition. Representative immunoblot shows the expression of DEK and HIF-1α. **B.** Cell proliferation assays were performed in HUVEC cells cultured in conditioned medium from MCF-7 cells stably transfected with DEK or empty vector under hypoxic condition and treated with a VEGF neutralizing antibody (α-VEGF). Representative immunoblot indicates the expression of DEK. *P < 0.05, **P < 0.01 (A, B). **C.** Wound healing assays were performed for HUVEC cells cultured in conditioned medium from MCF-7 cells stably infected as in A. **D.** Wound healing assays were performed for HUVEC cells cultured in conditioned medium from MCF-7 cells stably transfected and treated as in B. The image displayed is one of the representative results (C, D). Scale bar: 100 μm. All values shown are mean ± SD of triplicate measurements and have been repeated 3 times with similar results (C, D). *P < 0.05, **P < 0.01 versus empty vector or control shRNA plus empty vector.

Most types of cells, including tumor cells, but usually not endothelial cells themselves, secrete VEGF. Secreted VEGF plays critical roles in regulation of endothelial cell proliferation and migration [2–5]. Since DEK promotes VEGF secretion in breast cancer cells in HIF-1α-dependent and -independent manners, we tested the effect of the conditioned medium derived from knockdown DEK or DEK and HIF-1α knockdown stable breast cancer cell lines on HUVEC proliferation and migration. We performed cell proliferation assay and wound healing assay by incubating HUVEC cells with the condition medium derived from stable breast cancer cell lines. The conditioned medium from DEK knockdown MCF-7 and MDA-MB-231 cells inhibited HUVEC proliferation compared with control medium (Figure 3A; Supplementary Figure S3A). These effects could be rescued by the conditioned medium from these cells re-expressing DEK (Figure 3A; Supplementary Figure S3A). HIF-1α knockdown greatly reduced the ability of the conditioned medium from DEK knockdown cells to inhibit HUVEC proliferation. Neutralization of secreted VEGF by a VEGF neutralizing antibody abrogated the ability of the conditioned medium from DEK-overexpressing breast cancer cells to enhance HUVEC proliferation (Figure 3B; Supplementary Figure S3B), suggesting that DEK-mediated enhancement of VEGF expression in the conditioned medium is necessary for HUVEC proliferation. Similar trends were observed in HUVEC migration experiments (Figure 3C, 3D; Supplementary Figure S3C, S3D).

### Cancer cell-secreted VEGF modulated by DEK controls HUVEC tube formation and angiogenesis

**Figure 4:**
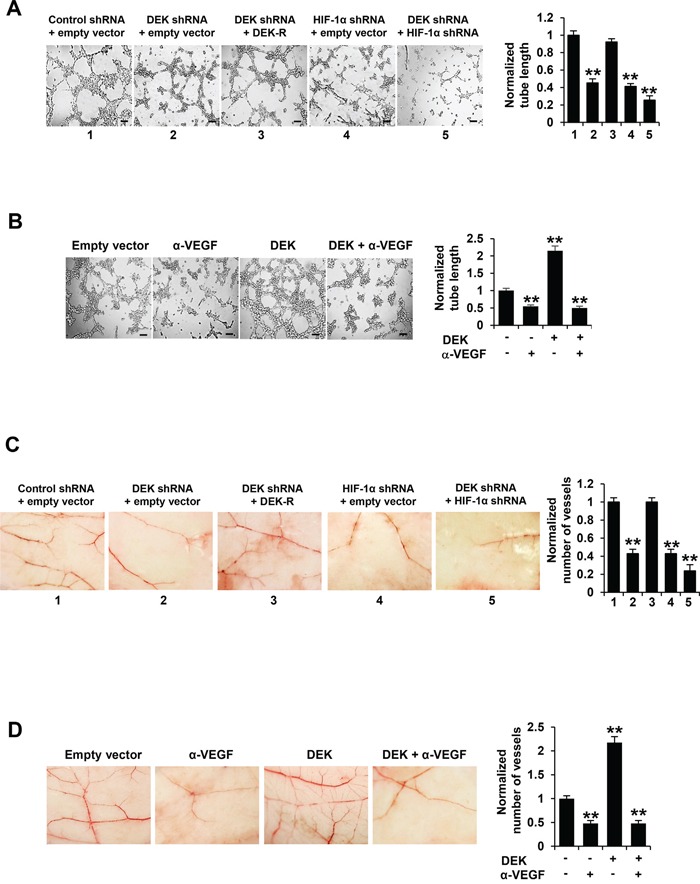
Cancer cell-secreted VEGF modulated by DEK regulates HUVEC tube formation and angiogenesis **A.** Tube formation assays were performed for HUVEC cells cultured in the conditioned medium from MCF-7 cells stably infected as in Figure 3A. **B.** Tube formation assays were performed for HUVEC cells cultured in the conditioned medium from MCF-7 cells stably transfected and treated as in Figure 3B. **C.** CAM assays were performed with conditioned medium from MCF-7 cells stably infected as in Figure 3A. **D.** CAM assays were performed with conditioned medium from MCF-7 cells stably transfected and treated as in Figure 3B. The image displayed is one of the representative results (A-D). Scale bar: 100 μm (A, B). All values shown are mean ± SD of triplicate measurements and have been repeated 3 times with similar results (A-D). *P < 0.05, **P < 0.01 versus empty vector or control shRNA plus empty vector. The raw data of tube formation and CAM was presented in Supplementary Table S2.

The key aspect of angiogenesis is the formation of capillaries from endothelial cells [39]. Thus, we first determined whether DEK-mediated VEGF expression affects HUVEC tube formation in *vitro*. We performed tube formation assay by incubating HUVEC cells with the condition medium derived from stable breast cancer cell lines. The conditioned medium from DEK knockdown breast cancer cells repressed HUVEC tube formation, a capillary-like structure with a lumen [38] (Figure 4A; Supplementary Figure S4A). The effects could be rescued by DEK reexpression in the DEK knockdown cells. HIF-1α knockdown greatly decreased the ability of the conditioned medium from DEK knockdown cells to inhibit HUVEC proliferation (Figure 4A; Supplementary Figure S4A). Inhibition of cancer cell-secreted VEGF by a VEGF neutralizing antibody abrogated the ability of the conditioned medium from DEK-overexpressing breast cancer cells to enhance HUVEC tube formation (Figure 4B; Supplementary Figure S4B).

Chick embryo chorioallantoic membrane (CAM) is one of the classical assays for studying angiogenesis in *vivo*. Therefore, we determined the effect of DEK-mediated VEGF expression on in *vivo* angiogenesis by CAM assay. CAM treated with the conditioned medium from DEK knockdown breast cancer cells had reduced number of new blood vessels and re-expression of DEK in DEK knockdown cells rescued this effect (Figure 4C; Supplementary Figure S4C). HIF-1α knockdown greatly reduced the ability of the conditioned medium from DEK knockdown cells to reduce the number of new blood vessels. Neutralization of cancer cell-secreted VEGF by a VEGF neutralizing antibody abolished the ability of the conditioned medium from DEK-overexpressing breast cancer cells to promote the formation of new blood vessels on the CAM (Figure 4D; Supplementary Figure S4D). Taken together, these data suggest that DEK-mediated enhancement of VEGF expression in the conditioned medium is responsible for HUVEC tube formation and angiogenesis.

### DEK is recruited to the regions containing DEK responsive element (DRE) and HRE of VEGF promoter and enhances the recruitment of HIF-1α and p300

To investigate the mechanism by which DEK enhances *VEGF* transcription in breast cancer cells, we first determined DEK binding sites on the VEGF promoter using luciferase reporter assay. We used a series of deletion constructs to map the binding sites of DEK. The deletion of −2304/−1128 bp had no effect on DEK-mediated enhancement of promoter reporter activity (Supplementary Figure S5A). The deletion of −1127/−728 bp decreased but not abolished DEK-mediated enhancement of promoter reporter activity, and further deletion of −727/−328 completely abolished DEK-mediated enhancement of promoter reporter activity (Supplementary Figure S5A), suggesting that DEK binding sites on *VEGF* promoter are located between −1127 and −728 bp and between −727 and −328 bp.

**Figure 5:**
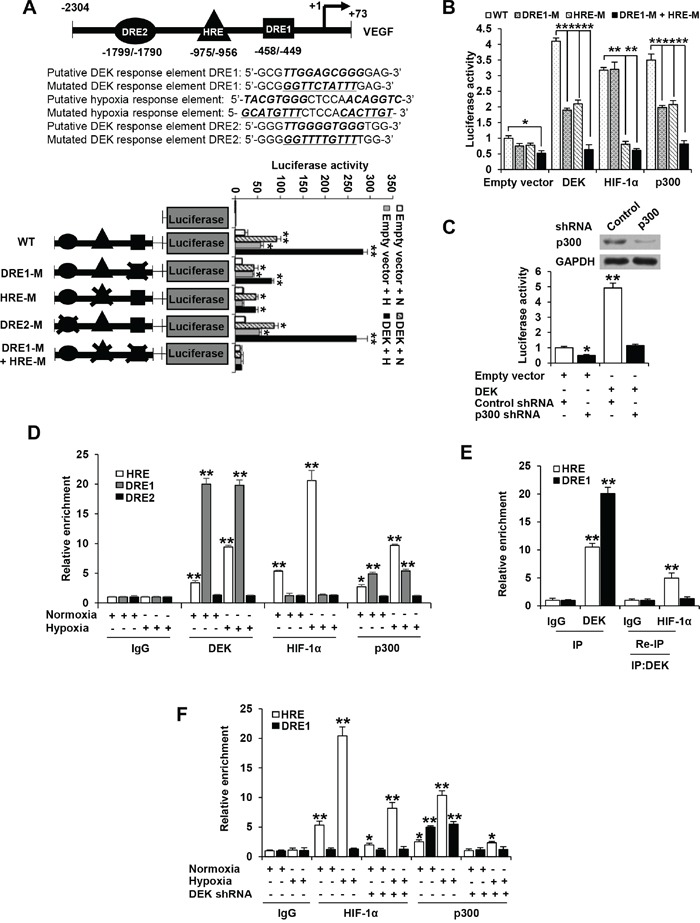
DEK is recruited to the DRE and HRE sites of VEGF promoter and promotes the recruitment of HIF-1α and p300 **A.** Luciferase activity of different VEGF promoter constructs in MCF-7 cells transfected with DEK or empty vector. At 24 h post-transfection, cells were exposed to either normoxic (N) or hypoxic (H) conditions for another 24 h. The arrow indicates the position of the transcriptional start site. DRE1 and DRE2 are putative DEK binding sites, and HRE is HIF-1α binding site. The “X” shows the mutated putative DEK-binding sites or HIF-1 binding site. **B.** Luciferase reporter assays were performed in MCF-7 cells cotransfected with indicated VEGF promoter constructs from A and DEK, HIF-1α or p300. The transfected cells were harvested for measurement of luciferase activities of different promoter constructs. **C.** Luciferase reporter assays were performed in MCF-7 cells cotransfected with the VEGF-Luc reporter and p300 shRNA. The transfected cells were harvested for measurement of VEGF-Luc activity. Representative immunoblot indicates the expression of p300. Data shown are mean ± SD of triplicate measurements and have been repeated 3 times with similar results. *P < 0.05, **P < 0.01 versus empty vector with corresponding promoter reporter (A-C). **D.** ChIP analysis of the occupancy of DEK, HIF-1α and p300 on DRE1, DRE2 or HRE of VEGF promoter in MCF-7 cells under normoxic or hypoxic conditions for 24 h. **E.** Re-ChIP analysis of the occupancy of DEK and HIF-1α on DRE1 and HRE of VEGF promoter in MCF-7 cells under hypoxic condition for 24 h. DEK formed a complex with HIF-1α on the HRE site but not on the DRE1 site. **F.** ChIP analysis were performed to detect the occupancy of HIF-1α and p300 on DRE1 and HRE of VEGF promoter in MCF-7 cells stably infected with lentivirus carrying DEK shRNA or control shRNA under normoxic or hypoxic conditions for 24 h. All values shown are mean ± SD of triplicate measurements and have been repeated 3 times with similar results (D-F). *P < 0.05, **P < 0.01 versus corresponding control (D-F).

DEK has been shown to bind TTGGTCAGGG motif [30]. Based on this motif and the above-mentioned deletion promoter reporter activity, there are two putative DEK binding sites on the *VEGF* promoter (from −2304 to +73 bp), one from −458 to −449 bp (DRE1) located between −727 and −328 bp and the other from −1799 to −1790 bp (DRE2). The HIF-1α binding site HRE (from −975 to −956 bp) is located between −1127 and −728 bp. We made the mutants by mutating pyrimidine nucleosides to purine nucleotides or purine nucleosides to pyrimidine nucleotides within the binding sites (Figure 5A). Consistent with the results of the reporter analysis with deletion constructs, the mutation of DRE1 or HRE site, but not DRE2, decreased DEK-mediated enhancement of promoter reporter activity under normoxic or hypoxic conditions (Figure 5A). The mutation of both DRE1 and HRE completely abolished DEK-mediated enhancement of promoter reporter activity (Figure 5A), indicating that the DRE1 and HRE sites are responsible for DEK modulation of VEGF promoter reporter activity.

The histone acetyltransferase p300 have been shown to be coactivators of many transcription factors, including HIF-1α [40, 41]. Since DEK enhances *VEGF* transcription partially dependent on HIF-1α and DEK modulation of VEGF transcription requires DRE1 and the HIF-1α binding site HRE, we investigated whether and how DRE1 and HRE affect the ability of HIF-1α and p300 to enhanced VEGF-Luc activity. As expected, HIF-1α and p300 increased the activity of the VEGF-Luc reporter containing HRE (Figure 5B), confirming that HIF-1α and p300 can enhance *VEGF* promoter activity via HRE. HRE mutation abolished the ability of HIF-1α to enhance VEGF-Luc activity but DRE1 mutation did not (Figure 5B). However, both HRE mutation and DRE1 mutation inhibited VEGF-Luc activity mediated by p300 in a similar way to DEK (Figure 5B). These data suggest that p300 or DEK increases VEGF-Luc activity via both DRE1 and HRE, and HIF-1α enhances VEGF-Luc activity only through HRE. Moreover, knockdown of p300 greatly inhibited DEK-mediated enhancement of VEGF-Luc activity, suggesting that p300 is responsible for DEK modulation of *VEGF* promoter activity (Figure 5C).

Consistent with the results of the promoter reporter mutation analysis, chromatin immunoprecipitation (ChIP) assay indicated that DEK and p300 were recruited to the regions containing DRE1 and HRE, and HIF-1α was only recruited to HRE (Figure 5D). DEK, HIF-1α and p300 could not be recruited to the region containing DRE2. Re-ChIP experiments showed that DEK formed a complex with HIF-1α on the HRE site but not on the DRE1 site (Figure 5E). Moreover, electrophoretic mobility shift assay (EMSA) showed that purified DEK protein directly bound to the DRE1 site, but not to the mutated DRE1, DRE2 or HRE site (Supplementary Figure S5B). The mutation of DRE1 and a 100-fold excess of unlabeled probe corresponding to the DRE1 site completely abolished the DNA-protein interaction (Supplementary Figure S5B). To further map the domain that mediates the DEK-DRE1 interaction, the EMSA assay was performed with the DEK deletion mutants (Supplementary Figure S5C). DEK(188-375) containing the second DNA binding domain mediated the interaction with DRE1, while DEK(1-187) containing the first DNA binding domain did not.

Since DEK knockdown decreased *VEGF* transcription, and HIF-1α and p300 are involved in this process, we examined whether DEK affects the recruitment of HIF-1α and p300 to the *VEGF* promoter using ChIP assay. Under normoxic or hypoxic conditions, DEK knockdown markedly inhibited the recruitment of HIF-1α to the HRE site and p300 to the HRE and DRE1 sites (Figure 5F).

### DEK interacts with HIF-1α and p300 and forms a complex with HIF-1α and p300

**Figure 6:**
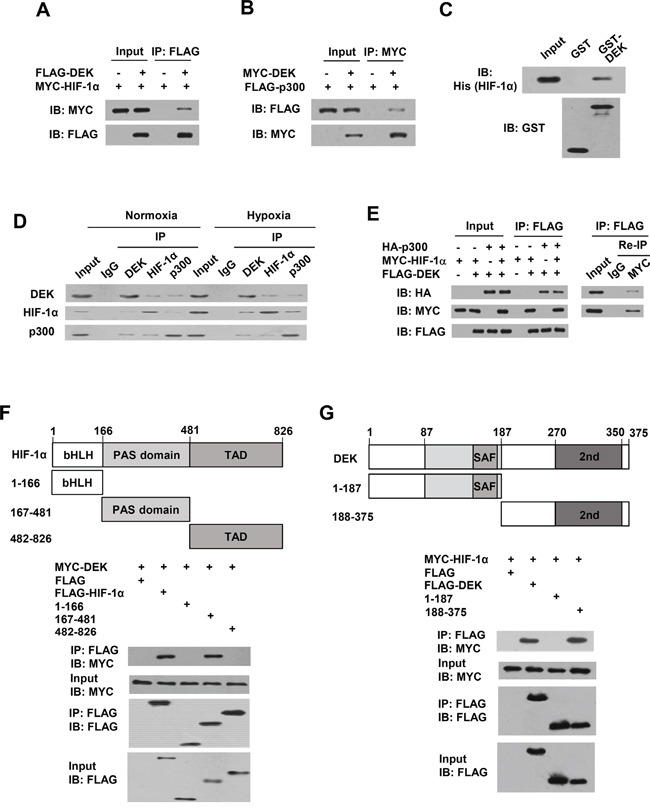
DEK, HIF-1α and p300 form a complex in vivo **A.** Co-IP assays with HEK293T cells cotransfected with MYC-tagged HIF-1α and FLAG-tagged DEK. Cell lysates were immunoprecipitated (IP) with anti-FLAG, followed by immunoblotting (IB) with the indicated antibodies. **B.** Co-IP assays with HEK293T cells cotransfected with MYC-tagged DEK and FLAG-tagged p300. Cell lysates were immunoprecipitated with anti-MYC, followed by immunoblotting with the indicated antibodies. **C.** GST pull-down analysis of direct interaction between DEK and HIF-1α. Purified His-tagged HIF-1α was incubated with purified GST-DEK or GST beads. Immunoblot with anti-His was performed. **D.** Reciprocal co-IP analysis of endogenous interactions among DEK, HIF-1α and p300 under normoxic or hypoxic conditions for 24 h. Immunoprecipitation were performed with antibodies against DEK, HIF-1α or p300, followed by immunoblot with the indicated antibodies. **E.** HEK293T cells were cotransfected with MYC-tagged HIF-1α, FLAG-tagged DEK and HA-tagged p300. Cell lysates were immunoprecipitated with anti-FLAG (1st IP). The immune complexes were eluted with FLAG peptide and re-immunoprecipitated (Re-IP) with anti-Myc or normal IgG, followed by immunoblotting with the indicated antibodies. **F.** HEK293T cells were cotransfected with MYC-tagged DEK and FLAG-tagged HIF-1α or its deletion mutants. Cell lysates were immunoprecipitated with anti-FLAG, followed by immunoblotting with the indicated antibodies. Schematic diagram of HIF-1α and its deletion mutants is shown. bHLH, basic-Helix-Loop-Helix; PAS, Per-ARNT-Sim; TAD, transactivation domain. **G.** HEK293T cells were cotransfected with MYC-tagged HIF-1α and FLAG-tagged DEK or its deletion mutants. Cell lysates were immunoprecipitated and analyzed as in A. Schematic diagram of DEK and its deletion mutants is shown. SAF, scaffold attachment factor (first DNA-binding domain); 2nd, second DNA-binding domain.

HIF-1α was shown to interact with p300 [41]. Based on our findings that DEK, p300 and HIF-1α are recruited to the HRE and DRE1 of the VEGF promoter, we tested whether DEK interacts with HIF-1α and p300. Co-immunoprecipitation (co-IP) assays showed that DEK interacted both with HIF-1α and with p300 in human embryonic kidney HEK293T cells (Figure 6A, 6B). The interaction between DEK and HIF-1α is direct because purified His-tagged HIF-1α protein interacted with purified GST-DEK protein but not GST alone (Figure 6C). However, DEK could not interact with HIF-1β (Supplementary Figure S6A). Importantly, reciprocal co-IP experiments demonstrated that endogenous DEK, HIF-1α, and p300 proteins interacted with each other in MCF-7 cells (Figure 6D). Co-IP combined with re-IP experiments indicated that DEK, HIF-1α and p300 formed a complex in MCF-7 cells (Figure 6E). Moreover, HIF-1α (167-481) containing the PER-ARNT-SIM (PAS) domain bound to DEK, whereas HIF-1α (1-166) and HIF-1α (482-826), containing the basic helix-loop-helix motif (bHLH) and the transactivation domain (TAD), respectively, did not (Figure 6F). DEK (188-375) containing the second DNA binding domain, but not DEK (1-187) containing the first DNA-binding domain, associated with HIF-1α (Figure 6G).

Since both DEK and HIF-1β bind the PAS domain of HIF-1α, we investigated whether DEK regulates HIF-1α and HIF-1β dimerization. Co-IP experiments indicated that DEK enhanced the interaction of HIF-1α and HIF-1β (Supplementary Figure S6B), suggesting that DEK does not compete with HIF-1β for binding HIF-1α. This enhancement may be explained by the possibility that DEK and HIF-1β interacts with different regions of the PAS domain. To this end, we mapped the interaction of DEK and HIF-1β in HIF-1α PAS domain. Indeed, DEK bound to PAS (300-481) (Supplementary Figure S6C) and HIF-1β bound to PAS (167-299) (Supplementary Figure S6D). Acriflavine is an agent that inhibits HIF-1α/HIF-1β dimerization by competing with HIF-1β for binding to HIF-1α PAS (235-299) [42]. We found that acriflavine inhibited HIF-1α and HIF-1β interaction but had no effect on interaction of HIF-1α with DEK (Supplementary Figure S6E).

### DEK modulates tumor angiogenesis and growth in HIF-1α-dependent and -independent manners

**Figure 7:**
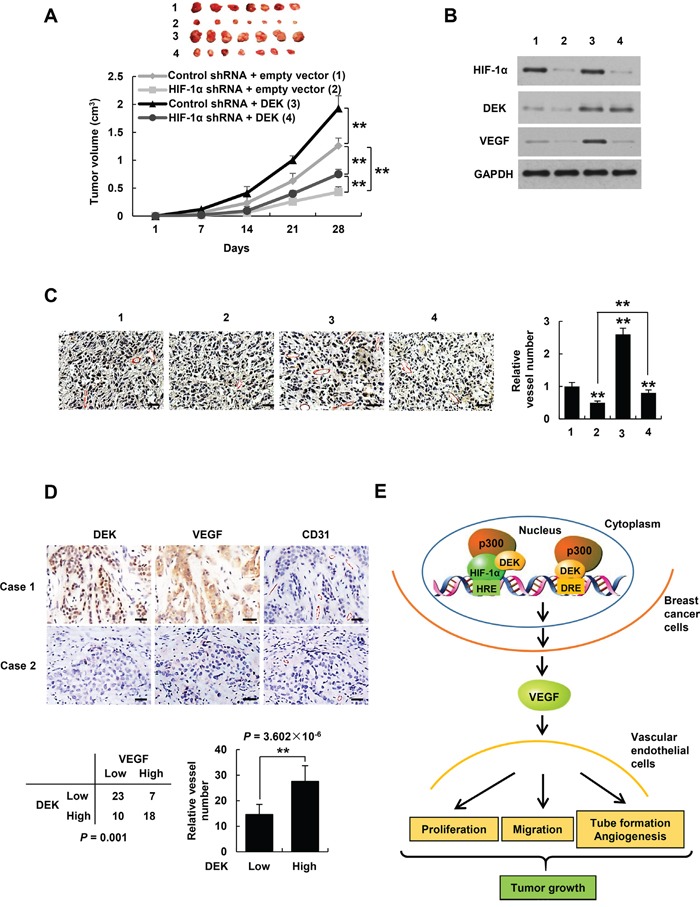
DEK controls tumor angiogenesis in HIF-1α-dependent and -independent manners **A.** Volume of xenograft tumors derived from MDA-MB-231 cells stably infected with lentiviruses carrying the indicated constructs. Data are shown as mean ± SD (n = 7). **P < 0.01 on day 28. **B, C.** Representative tumor tissues from A were subjected to immunoblot with the indicated antibodies (B) and immunohistochemical staining with an anti-CD31 antibody (C). Pictures from eight areas in each group were taken and the vessel number was manually counted (C). Scale bar: 50 μm. **D.** Representative immunohistochemical staining of DEK, VEGF and CD31 in human breast cancer samples. Scale bar: 25 μm. For the quantification of microvessel density, pictures from eight areas in each tissue were taken and the vessel number was counted. The correlation of DEK with VEGF or microvessel number (positive CD31 staining) is shown. The *P* value was generated using Pearson's χ2 test (DEK and VEGF) and Wilcoxon ranked sum test (DEK and CD31). **E.** Proposed model for DEK modulation of VEGF expression and tumor angiogenesis. DEK directly binds to the DRE of the VEGF promoter and indirectly binds to the HRE via its interaction with HIF-1α, and recruits the histone acetyltransferase p300 to both sites, resulting in increased VEGF transcription and secretion in breast cancer cells. The cancer cell-secreted VEGF promotes vascular endothelial cell proliferation, migration and tube formation as well as tumor angiogenesis and growth.

Cancer cells have been shown to produce VEGF not only for proliferation of endothelial cells but also for proliferation and/or survival of cancer cells in an autocrine and/or paracrine manner [43, 44]. Based on our findings that DEK enhances VEGF expression in HIF-1α-dependent and -independent manners, and DEK-enhanced VEGF controls HUVEC proliferation, migration and tube formation as well as angiogenesis in the chick chorioallantoic membrane, we tested the effects of DEK on tumor angiogenesis and growth, and the roles of HIF-1α in these processes. The nude mice were inoculated with MDA-MD-231-derived stable cell lines indicated in Figure 7A, and the tumors were taken out to detect the expression of VEGF and CD31 by Western blot and immunohistochemistry (IHC). The tumors in mice inoculated with DEK-overexpressing MDA-MD-231 cells grew faster than those inoculated with cells that did not overexpress DEK (Figure 7A). HIF-1α knockdown inhibited MDA-MB-231 tumor growth. HIF-1α knockdown reduced but not abolished the ability of DEK to promote MDA-MB-231 tumor growth. Western blot with VEGF antibody and IHC with an antibody against CD31, a vascular marker, showed that overexpression of DEK promoted VEGF expression and tumor angiogenesis, and HIF-1α knockdown inhibited but not abolished DEK-mediated enhancement of VEGF expression and angiogenesis (Figure 7B, 7C). Taken together, these data indicate that DEK enhances breast tumor growth and angiogenesis in HIF-1α-dependent and -independent manners.

### DEK positively correlates with VEGF expression and microvessel number in breast cancer patients

To explore correlation of DEK with VEGF expression and angiogenesis under pathological conditions, we conducted IHC of 58 human breast tumor samples with DEK, VEGF and CD31 antibodies. We confirmed the specificity of the antibody for DEK used in IHC by antigen competition and immunoblotting of lysates from ZR75-1 and MCF-7 cells infected with DEK shRNA1 (Supplementary Figure S7A, S7B). Consistent with previous reports [12], DEK was overexpressed in breast cancer patients (data not shown). Importantly, expression of DEK positively correlated with VEGF expression and microvessel number in 58 human breast cancer tissues (*P* = 0.001 and *P* = 3.602 × 10^−6^, respectively) (Figure 7D). These data implicate the significance of DEK in breast tumor angiogenesis.

## DISCUSSION

Angiogenesis is necessary for tumor growth and metastasis [1]. This process is mainly mediated by hypoxia-induced expression of VEGF [7–9]. Cellular hypoxia is a critical phenomenon in cancer. HIF-1α is a main mediator of hypoxia response and a key regulator of VEGF expression and angiogenesis. The transactivation of HIF-1α requires the recruitment of coactivators such as p300/CBP [46]. Like HIF-1α, p300 can also enhance angiogenesis [47]. Thus, disruption of the HIF-1α-p300 complex is a means of inhibiting HIF1α activity and angiogenesis. We show that DEK knockdown reduces recruitment of HIF-1α and p300 to the VEGF promoter and the transcription of VEGF, suggesting that DEK is a key coactivator for HIF-1α. On the other hand, DEK can induce VEGF transcription in HIF-1α-independent manner. Importantly, we demonstrate that DEK promotes tumor angiogenesis and growth in HIF-1α-dependent and -independent manners. Moreover, DEK expression positively correlates with the expression of VEGF and microvessel number in breast cancer patients, indicating the clinical relevance.

DEK plays roles in DNA supercoiling, DNA replication, RNA splicing and transcription [27–30]. DEK is also implicated in many cellular functions, including proliferation, differentiation, apoptosis, and senescence [30–33]. In this study, we demonstrate a novel function for DEK in tumor angiogenesis. DEK is overexpressed in multiple cancers, including breast cancer and prostate cancer. DEK can be transcriptionally activated by the E7 oncogene and transcription factors E2F and YY1 [33, 17, 48]. This transcriptional regulation may be involved in DEK overexpression in cancer. However, the exact mechanisms of DEK overexpression need to be further studied. The fact that DEK is overexpressed in many cancers, high DEK expression is associated with poor clinical outcome and DEK promotes tumor angiogenesis implicates the importance of DEK in cancer.

DEK contains two DNA-binding domains but does not have transactivation or transrepression domain [26–30]. DEK has been shown to act as a cofactor regulating transcriptional activity. DEK is a coactivator of the transcription factors CCAAT/enhancer binding protein-α (C/EBP-α), activator protein 2α (AP-2α) and ecdysone receptor (EcR) [49–51]. However, whether DEK binds to sequence-specific DNA is controversial. Some studies show that the binding of DEK to DNA is structure-specific but not sequence-specific [52–54]. However, two research groups demonstrate that DEK can bind to sequence-specific DNA. Markovitz and colleagues showed that DEK directly binds specifically to the TG-rich element (TTGGTCAGGG) in the enhancer of human immunodeficiency virus-2 (HIV-2) [30]. This research group also demonstrated that DEK binds in a sequence-specific manner to the Y-box motifs (CTAATTGGCC) in the promoter regions of several class II MHC genes [55]. Very recently, Lohmann *et al*. have reported that DEK interacts with an upstream enhancer element (TCTAGCTGGCCTGGGCCC) of the erythroid Krüppel-like factor (EKLF) promoter [56]. Using EMSA and ChIP assays, we observed that DEK binds to the 10-bp DNA fragment (TTGGAGCGGG) of the VEGF promoter. The sequence that DEK binds to in the VEGF promoter is similar to those DEK binds to in the HIV-2 and EKLF enhancers. Thus, based on our results and the previously reported DEK binding sequences, we propose that the specific DNA-binding motif for some DEK target genes is TGGXXXGGG, where X represents any single nucleotide.

Our observation that DEK enhances VEGF transcription by directly binding to the DRE of the VEGF promoter and indirectly binding to the HRE upstream of the DRE through its interaction with HIF-1α indicates that DEK acts as both a transcription factor and a transcriptional cofactor. GST pull-down and coimunoprecipitation showed that DEK physically interacts with HIF-1α. Reciprocal coimmunoprecipitation and re-coimmunoprecipitation experiments demonstrated that DEK, HIF-1α, and p300 interact with each other, and DEK, HIF-1α, and p300 can form a complex. Although DEK and p300 can be recruited to both the DRE and the HRE sites, HIF-1α can be recruited only to the HRE site (Figure 7E). This may be explained by the fact that different domains of HIF-1α interact with DEK and HRE. HIF-1α (1-166) containing bHLH has been shown to bind to HRE. We showed that HIF-1α (167-481) containing the PAS domain interacts with DEK. However, DEK(188-375) containing the second DNA-binding domain associates with both HIF-1α and DRE. Thus, the binding of DEK to DRE may hinder interaction of HIF-1α with DEK. It will be interesting to identify more DEK target genes enhanced by its direct binding of the target gene promoters/enhancers.

## MATERIALS AND METHODS

### Plasmids, shRNAs, lentiviral vectors and reagents

The eukaryotic expression vectors for FLAG-tagged DEK, HIF-1α, HIF-1β and p300, MYC-tagged DEK and HIF-1α, and HA-tagged p300 were constructed by inserting PCR-amplified fragments into pcDNA3 (Invitrogen) harboring FLAG, MYC, or HA tag at the amino terminus. Plasmids encoding GST or His fusion proteins were generated by cloning PCR-amplified sequences into pGEX-KG (Amersham Pharmacia Biotech) or pET-28a (Novagen). The VEGF promoter luciferase reporters were made by inserting PCR-amplified promoter fragments from genomic DNA into the pGL4-Basic vector (Promega). The cDNA target sequences of short hairpin RNAs (shRNAs) for DEK, HIF-1α and p300 are listed in Supplementary Table S1A. For lentiviral vector construction, shRNAs were cloned into pSIH-H1-puro (System Biosciences), and DEK or HIF-1α cDNA was inserted into pCDH plasmid (System Biosciences) according to the manufacturers' protocols.

Anti-MYC (sc-40HRP), anti-HA (sc-7392HRP) and anti-p300 (sc-585) antibodies were purchased from Santa Cruz Biotechnology; anti-FLAG (A8592), anti-FLAG M2 agarose (A2220), anti-HIF-1α (H6536) and anti-GAPDH (G9295) were obtained from Sigma-Aldrich; anti-CD31 (ab28364), anti-DEK (ab166624) and anti-p300 (ab10485) were purchased from abcam; anti-GST (RPN1236) and anti-His (27471001) antibodies were purchased from GE Healthcare Life Sciences; anti-VEGF (AF-293-NA) and anti-VEGF (MAB293-500) were purchased from R&D Systems; anti-DEK (610948) was purchased from BD Transduction Laboratories; and anti-HIF-1α (20960-1-AP) was purchased from Proteintech.

### Cell culture, stable cell lines, transfections and reporter assays

MCF-7, ZR75-1, MDA-MB-231, HEK293T cells were routinely cultured in DMEM (Invitrogen) containing 10% FBS (Hyclone). Stable cell lines were generated by lentiviral transduction using pSIH-H1-puro or pCDH plasmid (System Biosciences) as described previously [57]. Lipofectamine 2000 reagent was used for transfections following the manufacturer's protocol (Invitrogen). Luciferase reporter assays using VEGF-Luc reporter were performed as described previously [58].

### Hypoxia treatments

Cells were cultured in a hypoxic chamber (Billups-Rothenberg, Dell Mar, CA) flushed with a gas mixture consisting of 1% O_2_, 5% CO_2_, and 94% N_2_, and incubated for 24 h at 37°C in a humidified atmosphere. The cells were harvested inside the chamber.

### Screening for transcription factors that regulate VEGF promoter luciferase activity

A high-throughput assay based on reverse transfection of 704 transfection-ready cDNA plasmids from Transcription factor GFC-Transfection Array was used according to the manufacturer's instructions (Origene). Briefly, the Turbofectin 8.0 reagent (Origene), 100 ng of the VEGF-Luc reporter, and 100 ng of β-galactosidase reporter were added to each well of 384-well plates containing 60 ng of distinct cDNA plasmids. Complex formation was allowed for 20 min at room temperature before the addition of ZR75-1 cells (7500 cells/well). After a 48 h incubation, cells were harvested and analyzed for luciferase and β-galactosidase activities as described previously [58].

### Real-time reverse transcription-PCR (RT-PCR)

Total RNA was extracted from cell cultures by using the TRIzol reagent according to the manufacturer's protocol (Invitrogen). RNA was reverse transcribed into cDNA by Quantscript RT Kit (Tiangen). RT-PCR was performed by using primers derived from the human VEGF sequence with SYBR-green dye. The experiment group mRNA levels were calculated as 2^−Δ(ΔCt)^, where ΔCt = Ct_experiment_ - Ct_actin_ and Δ(ΔCt) =ΔCt_experiment_ –ΔCt_control_. The primer sequences are listed in Supplementary Table S1B.

### Enzyme-linked immunosorbent assay (ELISA) for human VEGF protein expression

Cells were seeded into 12-well plates. Forty eight hours later, the level of VEGF in the supernatant was determined using an ELISA kit for human VEGF according to the manufacturer's instructions (R&D Systems).

### Cell proliferation assay

HUVEC cells were seeded into 96-well plates and treated with conditioned medium for indicated times. Cell proliferation was examined by the CCK-8 Kit (Dojindo Laboratories) according to the manufacturer's instructions. The CCK-8 assay was based on the dehydrogenase activity detection in viable cells, WST-8 produced a water-soluble formazan dye upon reduction in the presence of an electron carrier and the amount of the formazan dye generated by the activity of dehydrogenases in cells was directly proportional to the number of living cells. Then, the absorbance at 450 nm was measured using a microplate reader, which represented the number of cells.

### Wound healing assay

Cells seeded in 12-well culture plates were scratched via a white pipette tip and were treated with conditioned medium. Cell migration into the wound surface was monitored at various times. Quantitation was done by measuring the distance of the wound edge of the migrating cells from the start point to the migrated point in three independent experiments.

### Tube formation assay

A pre-chilled 96-well sterile plate was coated with 50 μl thawed extracellular matrix (ECM) gel solution, and then incubated at 37°C for 30 min to 1 h until the gel was solidified. A total of 1.5~3 × 10^4^ HUVEC cells were suspended in conditioned medium and seeded in each coated well. After incubation at 37°C for 4 to 18 h, cells were examined for capillary-like network formation and photographed using an inverted microscope. The tube length was measured using Image-Pro Plus.

### Chick chorioallantoic membrane (CAM) assay

Fertilized eggs were incubated at 37°C and 60% humidity for 10 days. A square window was made on the air sac to expose the CAM. A sterile 0.25 cm-diameter methylcellulose filter paper was placed on the CAM, and 100 μl of conditioned medium was added to the filter immediately. Three days later, CAMs were removed from the eggs, fixed with methanol: acetone (1:1, v/v) for 15 min, and photographed by Nikon D7000 camera (Nikon, Japan). The number of blood vessels around the filter papers within 1 mm was counted.

### Chromatin immunoprecipitation (ChIP) and re-ChIP

ChIP assay was performed using the Magna ChIP Assay Kit (Millipore) according to the manufacturer's instructions. For re-ChIP, complexes were eluted from the primary immunoprecipitation by incubation with 10 mM DTT at 37¼C for 30 min and diluted 1:50 in re-ChIP buffer (150 mM NaCl, 1% Triton X-100, 2 mM EDTA, 20 mM Tris-HCl, pH 8.1) followed by re-immunoprecipitation with the second antibodies. Real-time PCR was performed to detect relative occupancy. The primers used for real-time PCR are listed in Supplementary Table S1C.

### GST pull-down and coimmunoprecipitation assays

For GST pull-down assay, GST or His fusion proteins were expressed and purified according to the manufacturers' instructions (Amersham Pharmacia and Qiagen). GST pull-down and coimmunoprecipitation experiments were performed as described previously [59]. Proteins were analyzed by immunoblot with indicated antibodies.

### Electrophoretic mobility shift assay (EMSA)

DNA-protein binging assays were performed using LightShift Chemiluminescent EMSA Kit according to the manufacturer's instructions (Pierce). The sequences of the oligonucleotides used are listed in Supplementary Table S1D.

### Animal experiments

Animal studies were performed in accordance with protocols approved by the Institutional Animal Care and Use Committee at Beijing Institute of Biotechnology. Five million breast cancer cells were injected into the abdominal mammary fat pad of 6-week-old female nude mice. Tumor size was measured at indicated times using calipers. Tumor volume was calculated according to the following formula: volume = (longest diameter × shortest diameter^2^)/2.

### Clinical samples and immunohistochemistry

Fifty-eight cases of primary breast carcinomas and adjacent noncancerous tissues were obtained from Chinese PLA General Hospital, with the informed consent of patients and with the approval of the Institutional Review Committees of Chinese PLA General Hospital. Immunohistochemistry of formalin-fixed paraffin-embedded samples was performed as described previously [60]. Mouse anti-DEK (610948, BD Transduction Laboratories), mouse anti-VEGF (MAB293-500, R&D Systems), and rabbit anti-CD31 antibody (ab28364, Abcam) were used at dilutions of 1:200, 1:100, and 1:50 as the primary antibodies for IHC. DEK or VEGF score was generated by multiplying the percentage of stained cells (0-25%, 1+; 26%-50%, 2+; 51-75%, 3+; 76%-100%, 4+) by the intensity of the staining (low, 1+; medium, 2+, strong, 3+). We defined score ≤ 6 as low DEK or VEGF, and score > 6 as high DEK or VEGF.

### Statistical analysis

Data were analyzed for normality using a Shapiro-Wilk test. Normally distributed data sets were analyzed with two-tailed student's *t*-test for two comparisons or one-way ANOVA test with Bonferroni correction for multiple comparisons. Correlation between expression of DEK and VEGF was determined using Pearson's χ2 test. All statistical tests were two-sided. Statistical calculations were performed using SPSS 17.0. *P* values of less than 0.05 were considered statistically significant.

## SUPPLEMENTARY FIGURES AND TABLES


