# Supplementary material for: The DEK oncogene activates VEGF expression and promotes tumor angiogenesis and growth in HIF-1α-dependent and -independent manners
Source: Oncotarget. 2016 Mar 14;7(17):23740–56. doi: 10.18632/oncotarget.8060 (PMC5029660; doi:10.18632/oncotarget.8060)
Supplement: Supplementary file 1 [file oncotarget-07-23740-s001.pdf]

The DEK oncogene activates VEGF expression and promotes tumor angiogenesis and growth in HIF-1α-dependent and -independent manners

SUPPLEMENTARY FIGURES AND TABLES

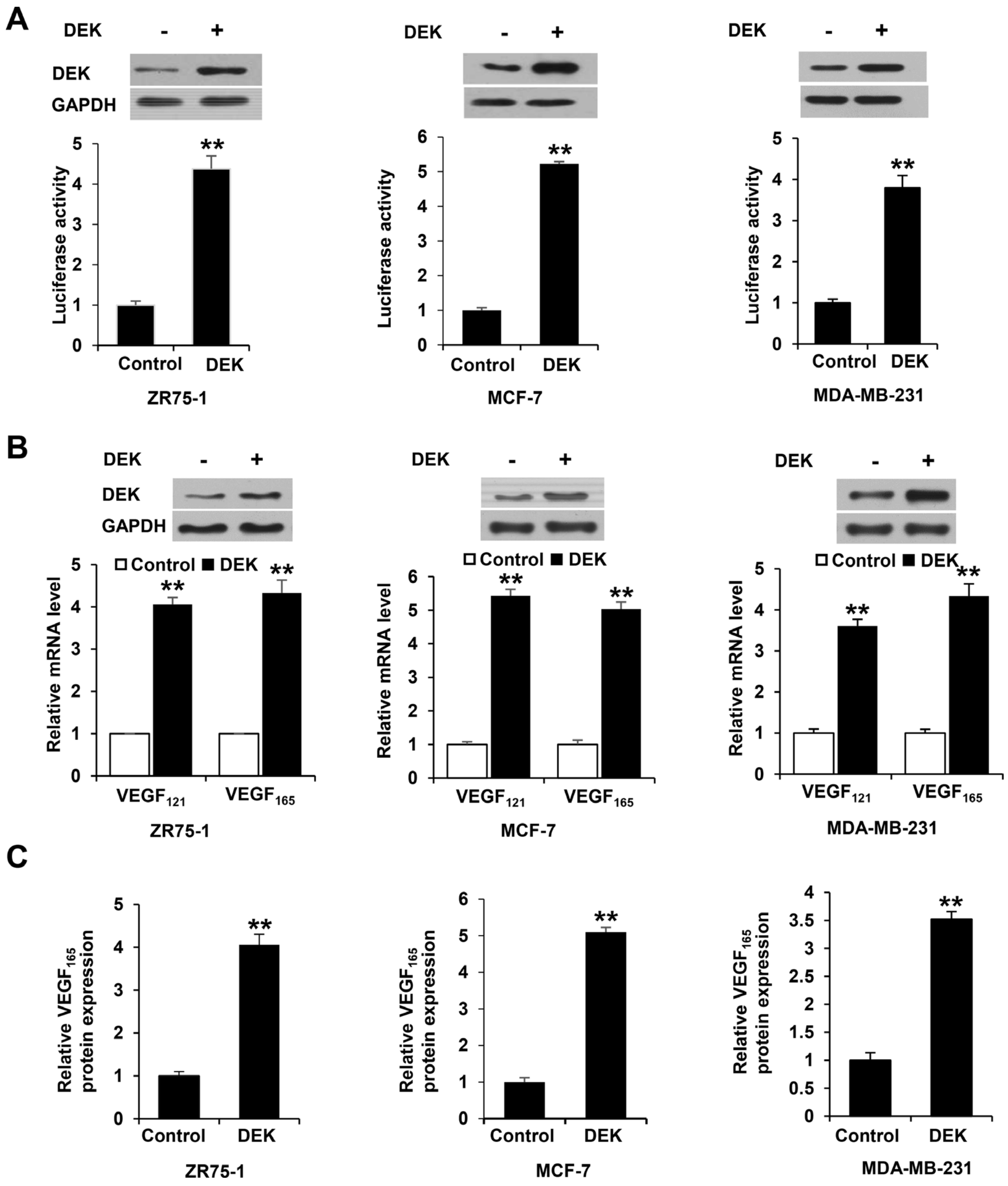

Supplementary Figure S1: DEK increases VEGF expression in breast cancer cells. A. Luciferase reporter assays in ZR75-1, MCF-7 and MDA-MB-231 breast cancer cells cotransfected with VEGF-Luc and DEK or empty vector (control). Representative immunoblot shows DEK expression. GAPDH was used as a loading control. **B.** Real-time RT-PCR analyses of the expression of $VEGF_{121}$ and $VEGF_{165}$, two major VEGF isoforms, in ZR75-1, MCF-7 and MDA-MB-231 cells stably infected with lentivirus carrying DEK or empty vector (control). Representative immunoblot indicates the expression of DEK. **C.** ELISA analyses of the VEGF concentration in cell supernatants from ZR75-1, MCF-7 and MDA-MB-231 cells stably infected as in B. Data shown are mean ± SD of triplicate measurements that have been repeated 3 times with similar results (A-C). $^{**}P < 0.01$ versus empty vector (control).

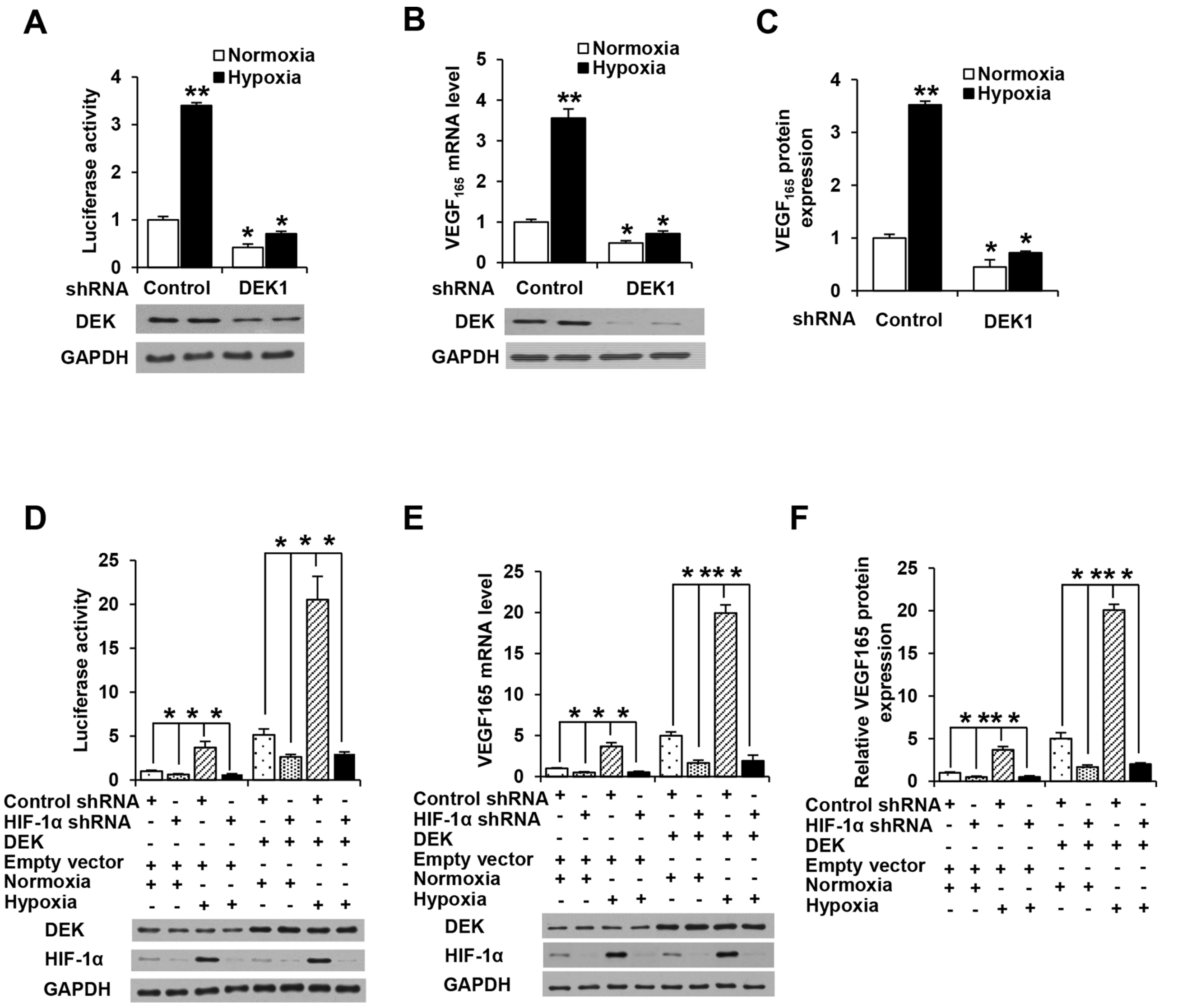

Supplementary Figure S2: DEK regulates VEGF expression in HIF-1α-dependent and -independent manners. A. Luciferase reporter assays in MDA-MB-231 breast cancer cells cotransfected with VEGF-Luc and DEK shRNA1 or control shRNA. Twenty four hours after transfection, cells were exposed to either normoxic (20% O_2) or hypoxic (1% O_2) conditions for another 24 h. Representative immunoblot indicates the expression of DEK. **B.** Real-time RT-PCR analysis of $VEGF_{165}$ expression in MDA-MB-231 cells stably infected with lentivirus carrying DEK shRNA1 or control shRNA. Cells were exposed to either normoxic or hypoxic conditions for 24 h before collected. Representative immunoblot shows the expression of DEK. **C.** ELISA analyses of the VEGF protein level in cell supernatants from MDA-MB-231 cells stably infected and treated as in B. **D.** Luciferase reporter assays in MDA-MB-231 cells cotransfected with VEGF-Luc, DEK and HIF-1α shRNA as indicated. Cells were treated and analyzed as in A. **E.** Real-time RT-PCR analyses of $VEGF_{165}$ expression in MDA-MB-231 cells stably infected with lentivirus carrying DEK and HIF-1α shRNA as indicated. Cells were treated and analyzed as in B. **F.** ELISA analyses of the VEGF protein level in cell supernatants from MDA-MB-231 cells stably infected and treated as in E. All values shown are mean ± SD of triplicate measurements and have been repeated 3 times with similar results (C, D). $^{*}P < 0.05$, $^{**}P < 0.01$ versus control shRNA or control shRNA plus empty vector.

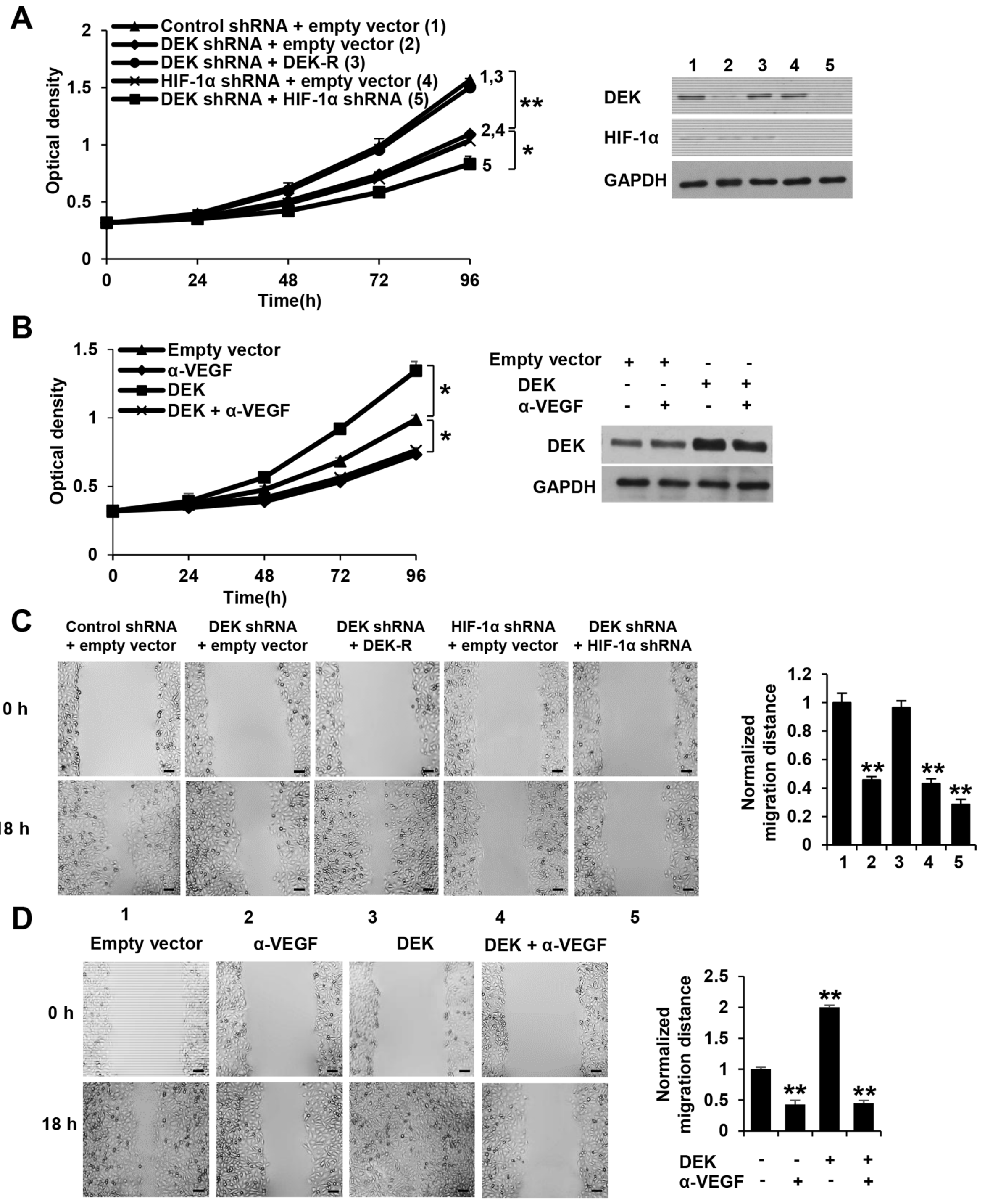

Supplementary Figure S3: Cancer cell-secreted VEGF regulated by DEK controls HUVEC proliferation and migration. A. Cell proliferation assays in HUVEC cells grown in conditioned medium from MDA-MB-231 cells stably infected with lentivirus harboring DEK shRNA, DEK shRNA plus DEK-resistant shRNA (DEK-R), HIF-1α shRNA or DEK shRNA plus HIF-1α shRNA under hypoxic conditions. Representative immunoblot indicates the expression of DEK and HIF-1α. **B.** Cell proliferation assays in HUVEC cells grown in conditioned medium from MDA-MB-231 cells stably transfected with DEK under hypoxic conditions and treated with a VEGF neutralizing antibody (α-VEGF). Representative immunoblot shows DEK expression. $^*P < 0.05$, $^{**}P < 0.01$ (A, B). **C.** Wound healing assays for HUVEC cells grown in conditioned medium from MDA-MB-231 cells stably infected as in A. **D.** Wound healing assays for HUVEC cells grown in conditioned medium from MDA-MB-231 cells stably transfected and treated as in B. The image displayed is one of the representative results (C, D). Scale bar: 100 µm. All values shown are mean ± SD of triplicate measurements and have been repeated 3 times with similar results (C, D). $^*P < 0.05$, $^{**}P < 0.01$ versus empty vector or control shRNA plus empty vector.

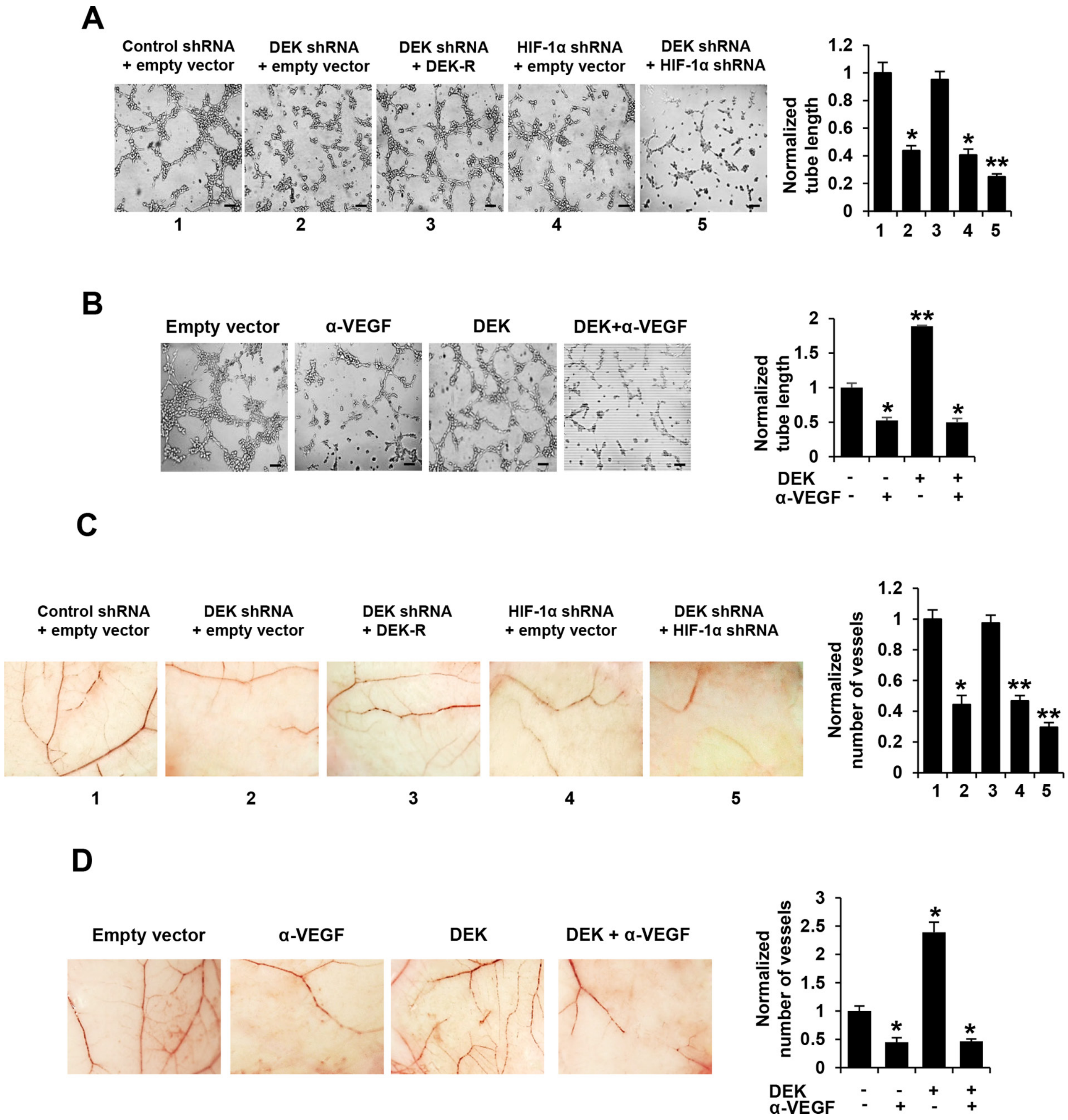

Supplementary Figure S4: Cancer cell-secreted VEGF regulated by DEK controls HUVEC tube formation and angiogenesis. A. Tube formation assays for HUVEC cells grown in the conditioned medium from MDA-MB-231 cells stably infected as in Supplementary Figure S3A. **B.** Tube formation assays for HUVEC cells grown in the conditioned medium from MDA-MB-231 cells stably transfected and treated as in Supplementary Figure S3B. **C.** CAM assays with conditioned medium from MDA-MB-231 cells stably infected as in Supplementary Figure S3A. **D.** CAM assays with conditioned medium from MDA-MB-231 cells stably transfected and treated as in Supplementary Figure S3B. The image displayed is one of the representative results (A-D). Scale bar: 100 μm (A, B). All values shown are mean ± SD of triplicate measurements and have been repeated 3 times with similar results (A-D). $*P < 0.05$, $**P < 0.01$ versus empty vector or control shRNA plus empty vector.

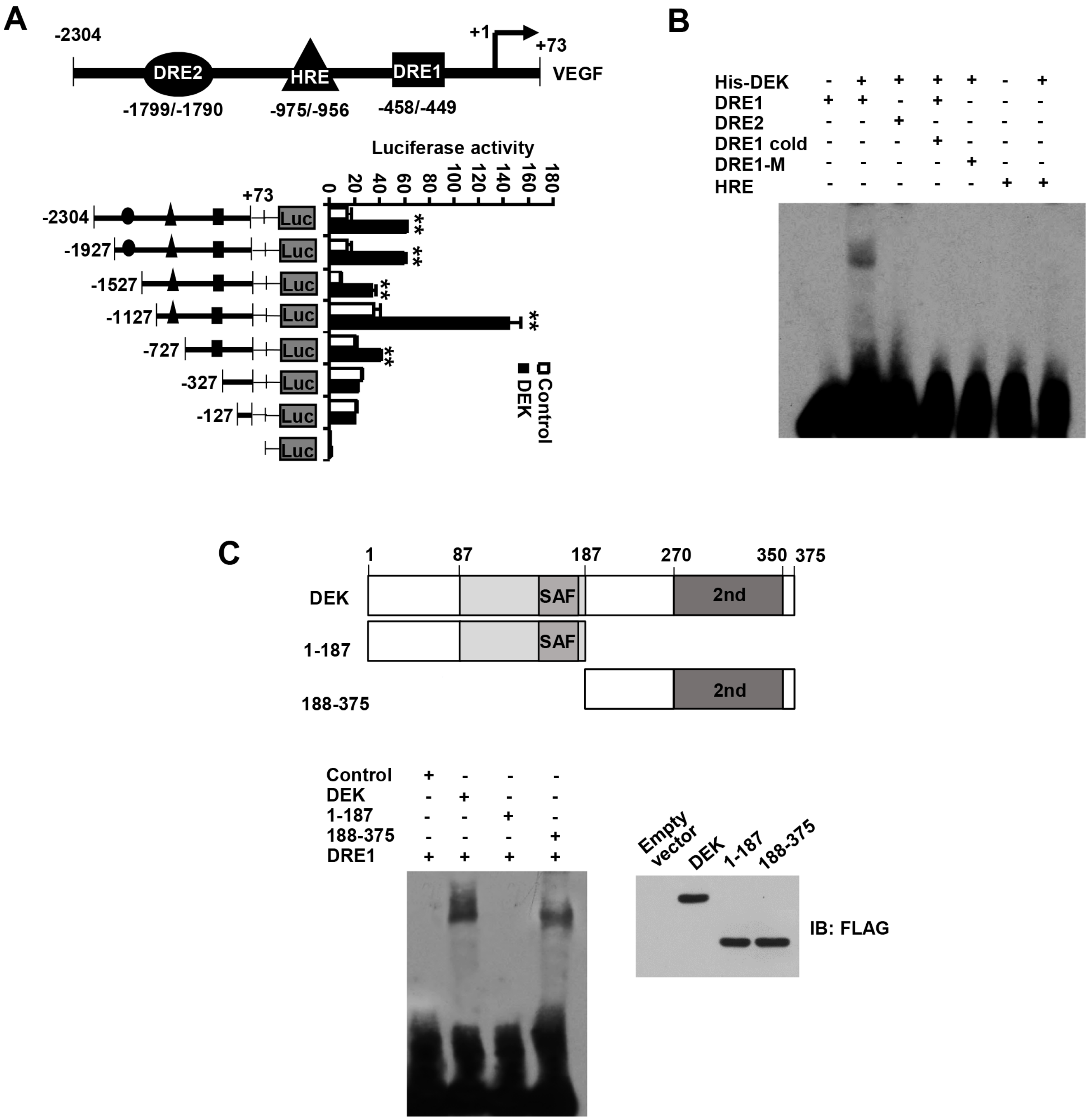

Supplementary Figure S5: DEK regulates VEGF promoter reporter activity and directly binds to the DRE1 site of the VEGF promoter. A. Luciferase activity of different VEGF promoter deletion constructs in MCF-7 cells transfected with DEK or empty vector (control). Data shown are mean ± SD of triplicate measurements and have been repeated 3 times with similar results. $^{**}P < 0.01$ versus empty vector with corresponding promoter reporter construct. **B.** EMSA assay of DEK binding site in the VEGF promoter. Purified His-tagged DEK protein was incubated with biotin-labeled probes containing the DRE1, DRE2, or HRE site, or mutated DRE1 site (DRE1-M) of the VEGF promoter, as indicated. For competition experiments, a 100-fold molar excess of unlabeled DRE1 was mixed with the biotin-labeled probe. **C.** EMSA assay of the DEK domain that binds to the DRE1 site of the VEGF promoter. Nuclear extracts from DEK knockdown MCF-7 cells transfected with empty vector (control), FLAG-tagged DEK or FLAG-tagged DEK deletion mutants were incubated with biotin-labeled probes containing the DRE1 site, as indicated. Western blot shows the expression of DEK and its deletion mutants with anti-FLAG. Schematic diagram of DEK and its deletion mutants is shown. SAF, scaffold attachment factor; 2nd DBD, second DNA binding domain.

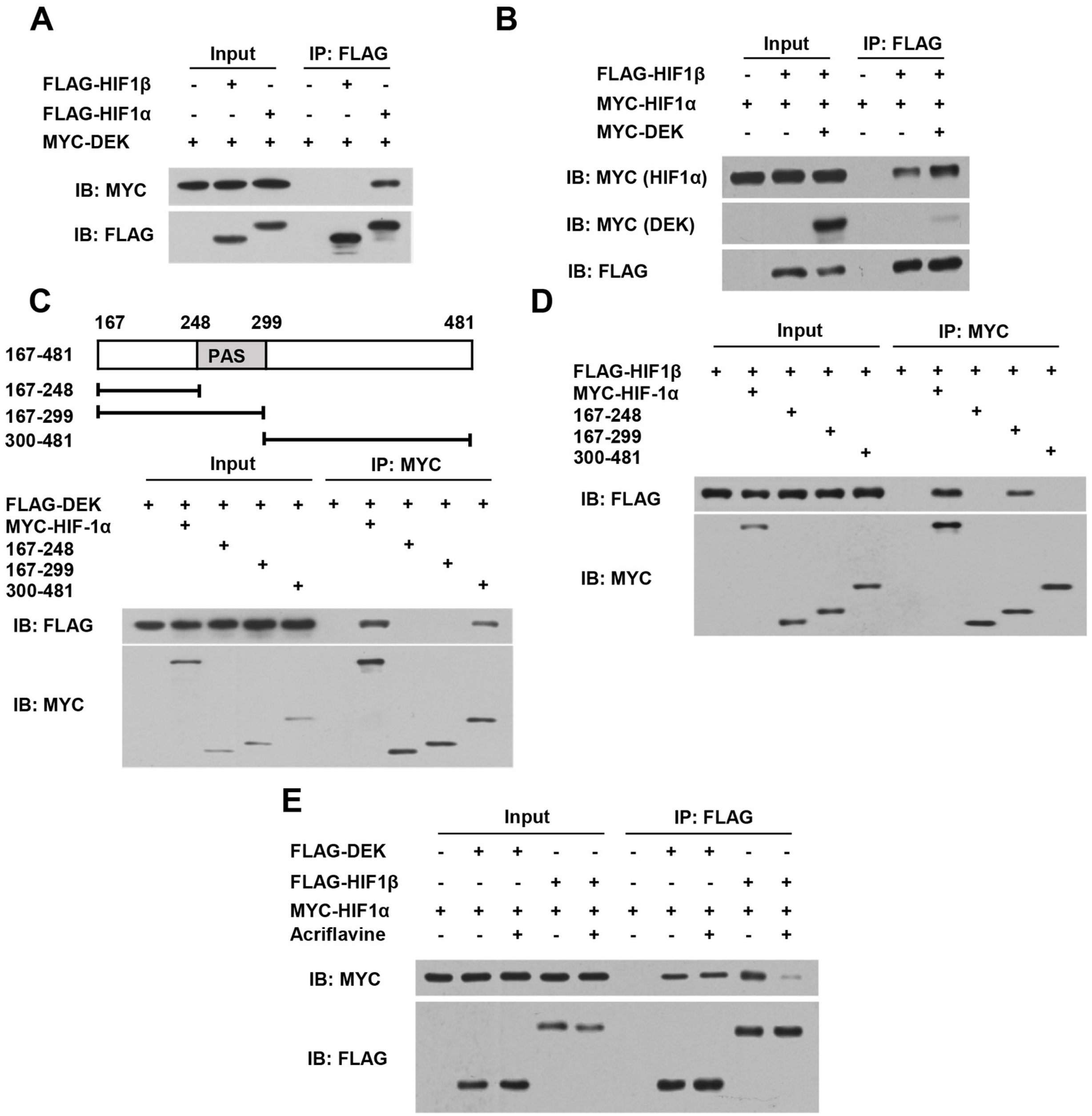

Supplementary Figure S6: DEK enhanced the dimerization of HIF-1α and HIF-1β. A. Co-IP assays with HEK293T cells cotransfected with FLAG-tagged HIF-1α or FLAG-tagged HIF-1β and MYC-tagged DEK. Cell lysates were immunoprecipitated (IP) with anti-FLAG, followed by immunoblotting (IB) with the indicated antibodies. **B.** Co-IP assays with HEK293T cells cotransfected with MYC-tagged HIF-1α and FLAG-tagged HIF-1β with or without MYC-tagged DEK. Cell lysates were immunoprecipitated (IP) with anti-FLAG, followed by immunoblotting (IB) with the indicated antibodies. **C.** HEK293T cells were cotransfected with FLAG-tagged DEK and MYC-tagged HIF-1α or its deletion mutants. Cell lysates were immunoprecipitated with anti-MYC, followed by immunoblotting with the indicated antibodies. Schematic diagram of HIF-1α and its deletion mutants is shown. **D.** HEK293T cells were cotransfected with FLAG-tagged HIF-1β and MYC-tagged HIF-1α or its deletion mutants. Cell lysates were immunoprecipitated with anti-MYC, followed by immunoblotting with the indicated antibodies. **E.** HEK293T cells were cotransfected with MYC-tagged HIF-1α and FLAG-tagged HIF-1β or FLAG-tagged DEK. Cell lysates were preincubated or did not preincubate with 5 μM acriflavine and immunoprecipitated with anti-FLAG, followed by immunoblotting with the indicated antibodies.

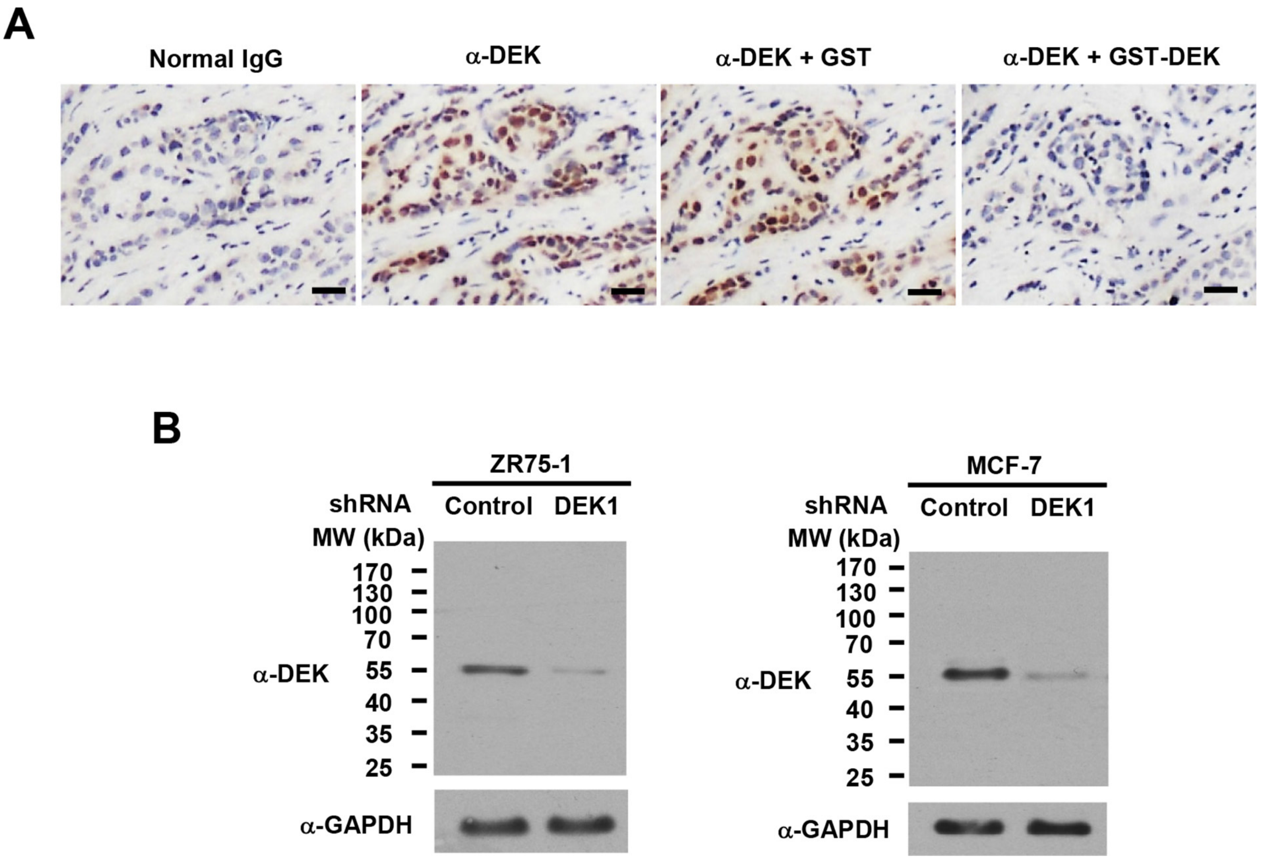

Supplementary Figure S7: Validation of antibody specificity to DEK. A. Representative immunohistochemical staining of breast cancer samples incubated with normal IgG or anti-DEK. To validate antibody specificity, the anti- DEK was pre-incubated with recombinant GST-DEK or GST for 1 h prior to applying to tissue. Scale bar, 25 µm. **B.** Immunoblot analysis of cell lysates from ZR75-1 (left panel) or MCF-7 (right panel) cells infected with control shRNA or DEK shRNA with antibodies specific for anti- DEK.

Supplementary Table S1: Sequences for shRNAs, RT-PCR, ChIP and EMSA

Supplementary Table S1A: The cDNA target sequences of shRNAs

Gene	Target sequence (5'→3')
DEK1	GGATAGTTCAGATGATGAACC
DEK2	TGTCCTCATTAAAGAAGAAGA
p300	CAATGAGTCACAGTCCTTTGAT
HIF-1α	AGGACAGTCACAAACAGGA

Supplementary Table S1B: Primers used for real-time RT-PCR

Gene	Forward (5'→3')	Reverse (5'→3')
DEK	AGGAGGAAGAGGACGAGGAC	GGAAAGCCACTGAACTGACC
$VEGF_{121}$	ATAGAGCAAGACAAGAAAAATG	ATCGTTCTGTATCAGTCTTTCCT
$VEGF_{165}$	AGAGCAAGACAAGAAAATCC	TACAAACAAATGCTTTCTCC

Supplementary Table S1C: Primers used for ChIP

VEGF Promoter	Forward (5'→3')	Reverse (5'→3')
HRE	CCTCTGTCTGCCCAGCTGCC	GTGGAGGTGCTAGGTTACC
DRE1	GTCTCTGGACAGAGTTTCC	CCTCAGCCCTTCCACACG
DRE2	CTAACTGTACAAAGACCTTG	GTGGAGGTGCTAGGTTACC

Supplementary Table S1D: Sequences of the oligonucleotides used for EMSA

Probe	Forward (5'→3')	Reverse (5'→3')
HRE	ACAGTGCATACGTGGGCTCCAACAG GTCCTCTTCCCTCCC	GGGAGGGAAGAGGACCTGTTGG AGCCCACGTATGCACTGT
DRE1	GGGTTGAGGGCGTTGGAGCGGGG AGAAGGCCAGGGGT	ACCCCTGGCCTTCTCCCCGCT CCAACGCCCTCAACCC
DRE1-M	GGGTTGAGGGCGGGTTCTATT TGAGAAGGCCAGGGGT	ACCCCTGGCCTTCTCAAAT AGAACCCGCCC TCAACCC
DRE2	TGGAAAGGGAGGGTTGGGGTG GGTGGGAGCCAGCCCT	AGGGCTGGCTCCCACCCA CCCCAACCCTCCCTTTCCA

Supplementary Table S2: The raw data of tube formation and CAM in Figure 4

Supplementary Table S2A: The raw data in Figure 4A

Experiment	Tube length (pixels)				
	1	2	3	4	5
1st	628.62	315.93	584.32	283.43	142.66
	674.21	274.24	618.19	253.72	188.52
2nd	699.46	338.89	690.07	317.21	159.69
	745.71	307.10	660.44	284.70	210.99
3rd	657.85	300.27	582.47	267.25	140.79
	588.08	271.61	621.10	233.61	160.10

Supplementary Table S2B: The raw data in Figure 4B

Experiment	Tube length (pixels)			
	1	2	3	4
1st	744.80	408.64	1458.3	326.20
	677.28	365.01	1596.2	380.21
2nd	656.79	387.79	1349.0	332.49
	708.68	349.56	1549.1	360.48
3rd	638.44	329.19	1258.1	322.33
	608.51	351.64	1474.5	344.16

Supplementary Table S2C: The raw data in Figure 4C

Experiment	Number of vessels				
	1	2	3	4	5
1st	10	5	10	4	2
	11	4	11	5	3
2nd	11	5	12	6	3
	13	6	10	6	4
3rd	12	6	11	5	3
	11	5	11	7	3

Supplementary Table S2D: The raw data in Figure 4D

Experiment	Number of vessels			
	1	2	3	4
1st	11	5	24	6
	12	6	26	5
2nd	10	4	20	5
	10	5	23	5
3rd	9	5	21	5
	11	4	23	4
